# The effects of fermentation products of prebiotic fibres on gut barrier and immune functions in vitro

**DOI:** 10.7717/peerj.5288

**Published:** 2018-08-10

**Authors:** Van T. Pham, Nicole Seifert, Nathalie Richard, Daniel Raederstorff, Robert Steinert, Kevin Prudence, M. Hasan Mohajeri

**Affiliations:** R&D Human Nutrition and Health, DSM Nutritional Products Ltd., Basel, Switzerland

**Keywords:** Prebiotics, Leaky gut, Tight junctions, Epithelial barrier, Oat β-glucan

## Abstract

The beneficial effects of prebiotic fibres on human health have been related to their capacities to alter the gut microbiota and modify the growth of beneficial microorganisms. It is long appreciated that bacterial metabolites affect the host’s physiology. The inner lining of the intestinal tract is the first level of interaction between the host and bacteria and their metabolites. Therefore, we set out to test the effects of five common dietary fibres (oat β-glucan 28%; oat β-glucan 94%; dried chicory root containing inulin 75%; xylo-oligosaccharide; inulin 90%) and maltodextrin, after fermentation by human gut microbiota in vitro, on measures of gut barrier integrity using a Caco-2/HT29-MTX co-culture as well as mucus production and immune parameters using HT29-MTX and HT29 cell models, respectively. Our data show that all fibres, fermentation products increased the tightness of the gut barrier with oat β-glucan 28% having the largest effect. Fermentation supernatants were tested also in models of the compromised gut barrier (leaky gut). After the addition of ethanol as basolateral stressor, only fermentation supernatant of oat β-glucan 28%, oat β-glucan 94% and maltodextrin improved the gut barrier integrity, while oat β-glucan 28% and dried chicory root containing inulin 75% significantly improved the gut barrier integrity after addition of rhamnolipids as apical stressor. Using the Luminex Technology, we demonstrated an important role of oat β-glucan fermentation products in modulating cytokine and chemokine productions. Furthermore, treating the goblet cells with effluent from xylo-oligosaccharide fermentation significantly increased mucus production. In summary, our data emphasize the potential positive effects of fermentation supernatant of dietary fibres on gut-related physiological outcomes and show that prebiotic fibres may have promising potential to induce specific gut health benefits.

## Introduction

The gut microbiome has significant impact on the host’s wellbeing and has been implicated in various diseases (Goulet, 2015). Recent evidence also revealed the critical role of the human gut microbiota in the development of brain functions relating to stress, anxiety, depression, and cognition (Mohajeri et al., 2018). In healthy conditions, the gut microbes reach a symbiotic relationship with the human immune system and, thus play a fundamental role in regulating the host’s immunity (Belkaid & Hand, 2014). In turn, the host immune system ensures a balanced microbial composition, control bacterial overgrowth and response to the invasion of pathogenic bacteria or microbial products via the intestinal barrier (La Fata, Weber & Mohajeri, 2017).

The large intestinal layer of specialized epithelial cells that are linked by tight junction proteins, together with a mucus layer, serve as a barrier that separates the host’s mucosa milieu from the luminal environment. Major cells of the intestinal epithelium are enterocytes, which are responsible for absorption of nutrients, and goblet cells, which produce, store and secrete the mucin glycoproteins, the major constituents of the mucus (Noah, Donahue & Shroyer, 2011). The highest density of goblet cells is reached in the colon (Groschwitz & Hogan, 2009; Srikanth & McCormick, 2008). The mucus layer in the gastrointestinal tract provides several benefits such as being the substrate for colonization of the colon by the adhering bacteria and the supply of nutrients for bacterial growth of specific phylogenetic groups (Lievin-Le Moal & Servin, 2006). Moreover, the mucus layer is rich in antimicrobial peptides and immunoglobulin A and thus is important for the protection of the epithelial cell surfaces from insults via creating a physical barrier (Gill, Wlodarska & Finlay, 2011; McGuckin et al., 2011) and by enhancing epithelium integrity (Resta-Lenert et al., 2011). There is evidently a strong interaction between the intestinal barrier and the gut microbiota and cells of the immune system (Takiishi, Fenero & Camara, 2017). Increased permeability of the epithelial layer, termed ‘leaky gut,’ allows the translocation of bacteria, antigens and toxins from the lumen to the lamina propria into the blood stream, which may trigger both local and systemic immune responses (Mu et al., 2017). A compromised intestinal barrier function might cause the disruption of the symbiotic relationship between gut microbiota and the immune system and is associated with the development of diseases and disorders such as inflammatory bowel disease, irritable bowel syndrome (IBS) and obesity (Bischoff et al., 2014). The intestinal barrier also possesses immune protective function by removing infected and damaged cells during the renewal process (Bischoff et al., 2014). On the other hand, the permeability of the epithelial lining can be modulated by microbiota and their products. Inevitably, reversing leaky gut by the modulation of the gut microbiota via therapeutics such as probiotics and prebiotic has gained considerable interest in recent years.

The most commonly used prebiotics in human diet are fructo-oligosaccharides, galacto-oligosaccharides (GOS), lactulose, and inulin. Other non-digestible complex carbohydrates that could potentially modulate the gut microbiota include resistant starch, cellulose, xylan, mannan, xyloglucan, β-glucan, and pectin. The impact of such prebiotic fibres on the modulation of the gut microbiota has been reviewed recently (Dahiya et al., 2017; Gibson et al., 2017; Holscher, 2017; Umu, Rudi & Diep, 2017). While several studies have confirmed the ability of probiotics to reverse the leaky gut by enhancing the production of proteins constituting the tight junctions (La Fata, Weber & Mohajeri, 2017; Mu et al., 2017), the effect of prebiotic fibres on gut epithelial barrier has not been investigated in detail.

A study in obese mice showed an increase of Zonula occludens-1 (ZO-1) and occludin mRNA expression in the jejunum of mice treated with prebiotic (oligofructose) compared to mice treated with a non-prebiotic control diet. Furthermore, immunohistochemical staining of both proteins revealed a higher protein expression in prebiotic treated mice, suggesting that prebiotic feeding improved the tight junctions and gut permeability (Cani et al., 2009). A more recent study using Western style diet-fed mice demonstrated that administration of inulin improved colonic inner mucus layer barrier function by correcting mucus penetrability. Interestingly, the synbiotic combination of *Bifidobacterium longum* and inulin did not prevent penetrability of the inner mucus layer (Schroeder et al., 2018). In a randomized, double-blind crossover study of healthy young male volunteers, the lactulose–mannitol excretion ratio was significantly decreased in the inulin-enriched pasta group compared to the baseline and control pasta group. Moreover, the inulin group had significantly lower zonulin serum values and higher GLP-2 values compared with the baseline and control pasta group. These results showed that inulin preserves intestinal mucosal barrier function and therefore could be used in the prevention of gastrointestinal diseases and metabolic disorders (Russo et al., 2012). Additional in vitro experiments showed that GOS has the potential to increase mucosal barrier function by stimulation of intestinal goblet cells in vitro (Bhatia et al., 2015).

Short chain fatty acids (SCFAs), the major products of fermentation of prebiotic fibres by the gut microbiota, exert beneficial effects on epithelial barrier integrity in vitro and in vivo. Several in vitro studies investigating their impact on intestinal permeability showed that at low concentrations, butyrate induced a decrease in permeability in a Caco-2 and HT-29 cell lines (Mariadason, Barkla & Gibson, 1997; Peng et al., 2007). Furthermore, butyrate has been shown to enhance intestinal barrier function by increasing expression of tight junction proteins such as claudin-1 and ZO-1 (Bordin et al., 2004; Ohata, Usami & Miyoshi, 2005; Wang et al., 2012). In contrast, higher concentration of butyrate could increase permeability in a Caco-2 cell line and in rat (Mariadason, Barkla & Gibson, 1997; Peng et al., 2007). In a comparative study, Elamin et al. (2013) compared the effects of acetate and propionate to the one exerted by butyrate on parameter of gut barrier integrity in vitro. These authors suggest a protective role of these SCFAs on the intestinal barrier function. These data are in agreement with a recent study showing that Arabinoxylooligosaccharides and inulin modulate the gut barrier function and immune response (Van den Abbeele et al., 2018).

Here we aimed to further elucidate the beneficial impact of the fermentation products of these prebiotic fibres on intestinal and immune health. In vitro fermentation samples were obtained from a recent study that compared the impact of prebiotic fibres on gut microbiota composition, including oat β-glucan 28%, oat β-glucan 94%, inulin 90%, dried chicory root containing 75% inulin and xylo-oligosaccharides (XOS) samples (Carlson et al., 2017). Our main objective is to investigate the impact of these prebiotic fibres on the intestinal epithelial integrity and mucus production using in vitro batch fermentation coupled with co-culture of Caco-2 and HT29-MTX-E12 epithelial cells as well as on selected cytokine and chemokine production relevant to immune response.

## Material and Methods

### Faecal collection

Fresh faecal samples were obtained from three healthy volunteers (Donor 1: 26 years old, female, BMI 28.1; Donor 2: 25 years old, male, BMI 26.3; Donor 3: 22 years old, male, BMI 23.0) at University of Minnesota, Department of Food Science and Nutrition (Carlson et al., 2017). All donors were healthy subjects and consumed a regular Western diet. Exclusion criteria were variables known to affect the balance of the gut microbiota, including specific diets, any known gastrointestinal diseases, the use of antibiotic in the last year and the use of dietary supplements. The samples were collected according to the procedure described previously (Carlson et al., 2017). All data and samples collected were done in accordance with University of Minnesota policies and procedures, including ethical approval of the study (Carlson et al., 2017).

### Batch fermentation

Oat bran contains 28% oat β-glucan, 24% insoluble fibres, 23% protein, 9% starch, 5% lipids, 6% water, 4% ash (DSM Nutritional Products, Ltd., Kaiseraugst, Switzerland). Dried chicory root contains 70–75% inulin, 6–8% pectin, and 4–6% hemi/cellulose (WholeFibre, Inc., Pennington, NJ, USA). β-glucan contains 94% high viscosity β-glucan from oat flour (Megazyme, Inc., Bray, Ireland). 70% corn-derived XOS (AIDP, Inc., City of Industry, CA, USA), 90% inulin (Cargill, Inc., Minneapolis, MN, USA) and a fully digestible maltodextrin (Sigma-Aldrich, Buchs, Switzerland) were also included in the study.

The faecal samples were collected anaerobically, immediately homogenized in phosphate buffer solution (1:6 w/v) using a vortex. Obtained faecal slurry was combined with prepared reducing solution (2.52 g cysteine hydrochloride, 16 mL 1 N NaOH, 2.56 g sodium sulfide nonanhydride, 380 mL DD H_2_O) at a 2:15 ratio. Ten mL of the prepared faecal inoculum was inoculated into the fermentation flasks within 5 min post defecation (Carlson et al., 2017). All samples (1.0 g of oat β-glucan 28%, delivering approximately 0.28 g of oat β-glucan; and 0.5 g of all other fibres) were hydrated in 40 mL sterile trypticase peptone fermentation medium (McBurney & Thompson, 1987) for 12 h at 4 °C followed by 2 h at 37 °C. The fermentation medium contained (per litre): 2.49 g trypticase peptone, 1.00 g ammonium bicarbonate, 8.75 g sodium bicarbonate, 1.43 g anhydrous sodium phosphate, 1.55 g anhydrous potassium phosphate monobasic, 0.60 g magnesium sulphate, 0.12 mg resazurin, 1.12 mmol calcium chloride, 0.63 mmol manganous chloride, 0.15 mmol cobalt chloride, 0.04 mmol ferric chloride (McBurney & Thompson, 1987). Ten mL of the faecal slurry was inoculated to each serum bottle. The final concentration of oat β-glucan 28% and other fibres is 2% and 1% (w/v), respectively. Batch fermentation was carried out at 37 °C under anaerobic conditions as described previously (Carlson et al., 2017). Effluent samples were collected from each fermentation flask before (0 h fermentation) and after fermentation (24 h fermentation), sterilized by filtering through 0.22 μm filters and immediately frozen at −80 C for in vitro analysis in cell culture systems.

### Cell culture

Co-cultures of Caco-2 and HT29-MTX-E12 cells were used to assess the protective effect of the fermentation supernatants.

CaCo-2 ECACC 86010202 and HT29-MTX-E12 ECACC 12040401 cells (both from European Collection of Cell Cultures, Salisbury, UK) were both cultured at 37 °C, in atmosphere of 5% CO_2_ in Dulbecco’s Modified Eagle Medium (DMEM) medium supplemented with 4.5g/L D-Glucose, four mM L-Glutamine, one mM Sodium Pyruvate, 1% MEM Non-Essential Amino Acids, 50 μg/mL Gentamicin (Life Technologies Europe B.V., Zug, Switzerland) and 10% heat-inactivated fetal bovine serum (FBS) (Sigma-Aldrich, Buchs, Switzerland). Sub-confluent cells were trypsinized using 0.25% Trypsin/EDTA (Life Technologies Europe B.V., Zug, Switzerland) and passaged at a ratio of 1:5–1:10 twice a week. Cells between passages 55 and 85 were used for this study.

Cells were seeded at a density of 20,000 cells/well in a 7:3 ratio (Caco-2:HT29-MTX-E12) in Corning HTS Transwell-24 system PET membranes, 0.4 μM pore size, cell growth area 0.33 cm^2^/well (Corning BV, Amsterdam, Netherlands) and cultured as described above. Media was changed every second to third day.

### Barrier integrity measurement in healthy gut model

After 13 days in culture, initial transepithelial electrical resistance (TEER) readings were obtained using an EVOM^2^ Voltohmmeter (EVOM; World Precision Instruments, Berlin, Germany) equipped with STX100 Electrodes. TEER values correlate with the tightness of the confluent monolayer (Srinivasan et al., 2015).

**Figure 1:**
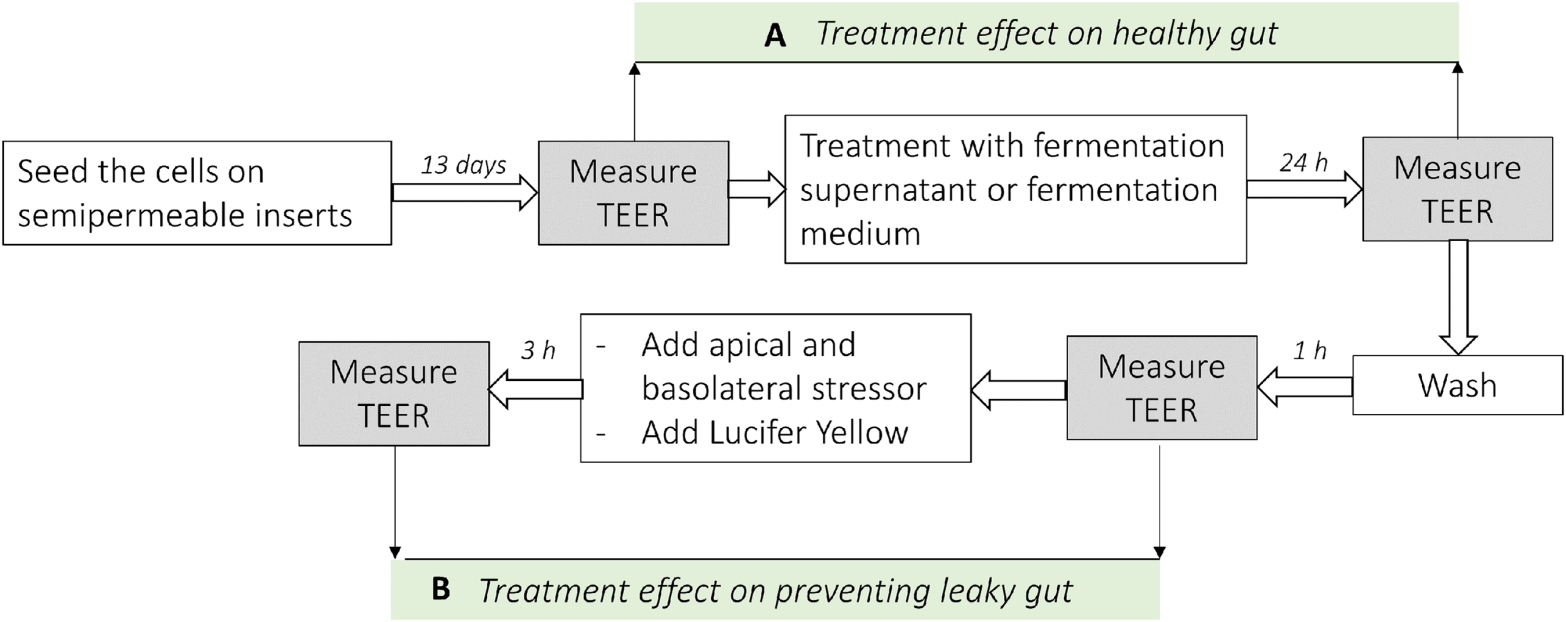
Analysis scheme used in co-culture cell model of the intestine. (A) Represents the difference of the TEER measurement after the treatment with fermentation supernatants without any stressor (Healthy gut model). (B) Represents the difference of the TEER measurement after the treatment with fermentation supernatants with the addition of apical or basolateral stressors (leaky gut model).

The background TEER (insert) was subtracted from total TEER (cell monolayer plus insert) to yield the monolayer resistance and then normalized to surface area by multiplying with the area of the insert (Srinivasan et al., 2015). The integrity of the monolayer was determined at basal level by measuring TEER. A TEER of 300 Ωcm^2^ was regarded as a tight monolayer (see Results section). Afterwards, the cells were treated with sterile filtered fermentation supernatants or fermentation medium as a control (diluted 1:20 in growth media) (Fig. 1). After 24 h incubation the resistance across each cell monolayer was measured again and the percentage change in TEER compared to initial TEER for each insert was calculated to represent a healthy gut model. All TEER measurements were done by placing the plates containing the inserts on a 37 °C warming plate and were performed in the same duration.

### Barrier integrity measurement in leaky gut model

The integrity of cell monolayers after the stressor test was evaluated by both TEER values and by measuring the transport of Lucifer yellow from apical to basolateral compartments (leaky gut model). A prewarmed Hank’s Balanced Salt Solution (HBSS) transport buffer composed of HBSS pH 7.4 with Ca^2+^ and Mg^2+^, containing 5.5 mM D-(+)-glucose, Sodium Bicarbonate, and supplemented with four mM L-glutamine, 20 mM HEPES (Life Technologies Europe B.V., Zug, Switzerland) was used for all permeability experiments. The inserts were rinsed twice with HBSS using a reservoir tray, 100 μL HBSS were then added to the apical site of the inserts and subsequently moved to new wells with fresh HBSS and allowed to equilibrate at 37 °C for 60 min in the incubator followed by TEER measurement to obtain baseline resistance readings.

Lucifer Yellow CH dilithium salt (Sigma-Aldrich, Buchs, Switzerland) was dissolved in HBSS at a final concentration of 100 μg/mL. Basolateral stressor EtOH (Merck, KGaA, Darmstadt, Germany) was diluted to 5% in HBSS. Apical Stressor Rhamnolipids R90 (AGAE Technologies, Corvallis, OR, USA) was dissolved in HBSS containing Lucifer Yellow at a final concentration of 350 μg/mL. All solutions were prepared freshly each time.

The HBSS transport buffer from the basolateral chamber was aspirated first, then the HBSS from the apical chamber and replaced with Lucifer yellow solution. For the apical stressor test, Lucifer yellow solution containing 350 μg/mL Rhamnolipids or Lucifer yellow solution without stressor for the control wells was loaded into the apical compartment, blank HBSS was subsequently added to the basolateral chamber. For the basolateral stressor test, Lucifer yellow solution was added to the apical side of all wells, and either 5% EtOH in HBSS or blank HBSS for the control wells was placed in the basolateral compartment.

After 3 h incubation at 37 °C the permeability of the cell monolayer was evaluated by measuring TEER values. TEER data were expressed as the percentage of the initial values.

After TEER measurements, 100 μL aliquot samples were withdrawn from the basolateral sites in duplicates to quantify the amount of Lucifer yellow transported. The fluorescence level (excitation at 420 nm and emission at 530 nm) was measured in a 96-well fluorescent plate reader (Spectramax M5 Series Multi-Mode Microplate Reader, Molecular Devices, Sunnyvale, CA, USA).

### Mucus staining with Alcian Blue

HT29-MTX-E12 cells were seeded in 96-well tissue-culture plates (Corning BV, Amsterdam, Netherlands) at an initial cell density of 40,000 cells/well in complete culture medium. Confluent (72 h post-seeding) cells were then incubated in growth media containing fermentation supernatants (1:40 diluted) or fermentation medium as a control.

After 4 day-incubation with fermentation supernatants, Alcian Blue staining was performed to detect acidic mucous substances in the monolayers (Behrens et al., 2001). In brief, cells were washed with Phosphate-buffered saline (PBS) and subsequently fixed with 4% paraformaldehyde (Thermo Scientific, Rockford, IL, USA) for 30 min at room temperature. Cells were washed with PBS and subsequently stained using the Alcian Blue pH 2.5 mucin stain kit (Abcam, Cambridge, UK) according to the manufacturer’s instructions. To this aim, the cells were pre-treated in Acetic Acid Solution for 3 min. Acidic Sulfated Mucosubstances and Sialomucins were stained with Alcian Blue for 30 min at room temperature. The plates were washed 2 min in running tap water, followed by two changes of distilled water. Absorbance at 600 nM in 100 μL distilled water was read with a Spectramax M5 Series Multi-Mode Microplate Reader (Molecular Devices, Sunnyvale, CA, USA).

Subsequently, nuclei were stained with 1μg/mL Hoechst 33342 solution (Life Technologies Europe, Bleiswijk, Netherlands) for 30 min at room temperature.

### Automated analysis of the cell number and normalization

Fourty-nine adjacent images (fields) of each well were acquired with a 10× objective using an ArrayScan VTI high-content screening system (Thermo Fisher Scientific, Pittsburgh, PA, USA), resulting in a field width of 640 microns. Channel one (Ch1) is the focus channel in which objects (Hoechst-stained nuclei) were identified. The cell numbers were determined by counting the fluorescent staining (Hoechst dye) and the mucus production was quantified by colorimetric analysis of Alcian Blue staining. The Alcian Blue absorbance was normalized to OD/100,000 cells.

### HT29 cell culture and quantification of immunological biomarkers

HT29 cells were obtained from American Type Culture Collection (Manassas, VA, USA). Cells were cultured in DMEM supplemented with 10% FBS, 50 units/mL penicillin, 50 μg/mL streptomycin, L-glutamine, and nonessential amino acids (Life Technologies Europe B.V., Zug, Switzerland). Cells were seeded into 12-well plates at 8 × 10^5^ cells per well and used after 2 days of preculture. They were starved in DMEM containing 0.25% FBS for 18 h before treatment. Cells were treated for 24 h with TNF-α at 100 ng/mL or fermentation supernatants which were sterile filtered and diluted 1:20 in DMEM containing 0.25% FBS. All treatments were done in triplicate. Consequently, cytokines, chemokines, and interleukins were quantified in HT29 supernatants using the Luminex Technology (LiquiChip Workstation IS 200, Qiagen, Hilden, Germany) with Bio-Plex Pro Human Cytokine Panel kits (Bio-Rad, Hercules, CA, USA) or with Luminex Screening Assay kits (R&D Systems, Inc., Minneapolis, MN, USA), following the manufacturer’s instructions. The data were acquired with the Luminex IS 2.3 software and evaluated with the LiquiChip Analyser software (Qiagen, Hilden, Germany).

### Statistical analysis

Statistical analyses were carried out with IBM SPSS Statistics 22.0 (IBM SPSS Inc., Chicago, IL, USA). TEER data were calculated as percentage change after treatment with the tested fibres and expressed as mean ± SEM. Lucifer yellow permeability was expressed as mean relative fluorescence units ± SEM. In healthy gut model, TEER values after the treatment with fermentation supernatants without any stressor were tested for normal distribution using Shapiro–Wilk test, and means were compared pairwise using Student’s *t*-test for normally distributed data. A non-parametric Mann–Whitney test was performed when data were not normally distributed. In the leaky gut model, TEER and relative fluorescence units were compared pairwise using Student’s *t*-test. Mucus production data were expressed as normalized Alcian Blue absorbance ± SD and were compared using two tailed Student’s *t*-test. Cytokines and chemokines concentration were expressed as pg/mL ± SD and were compared using two tailed Student’s *t*-test. *P*-values < 0.05 were considered significant.

## Results

### Effects of fibres on gut barrier integrity using healthy gut model

**Figure 2:**
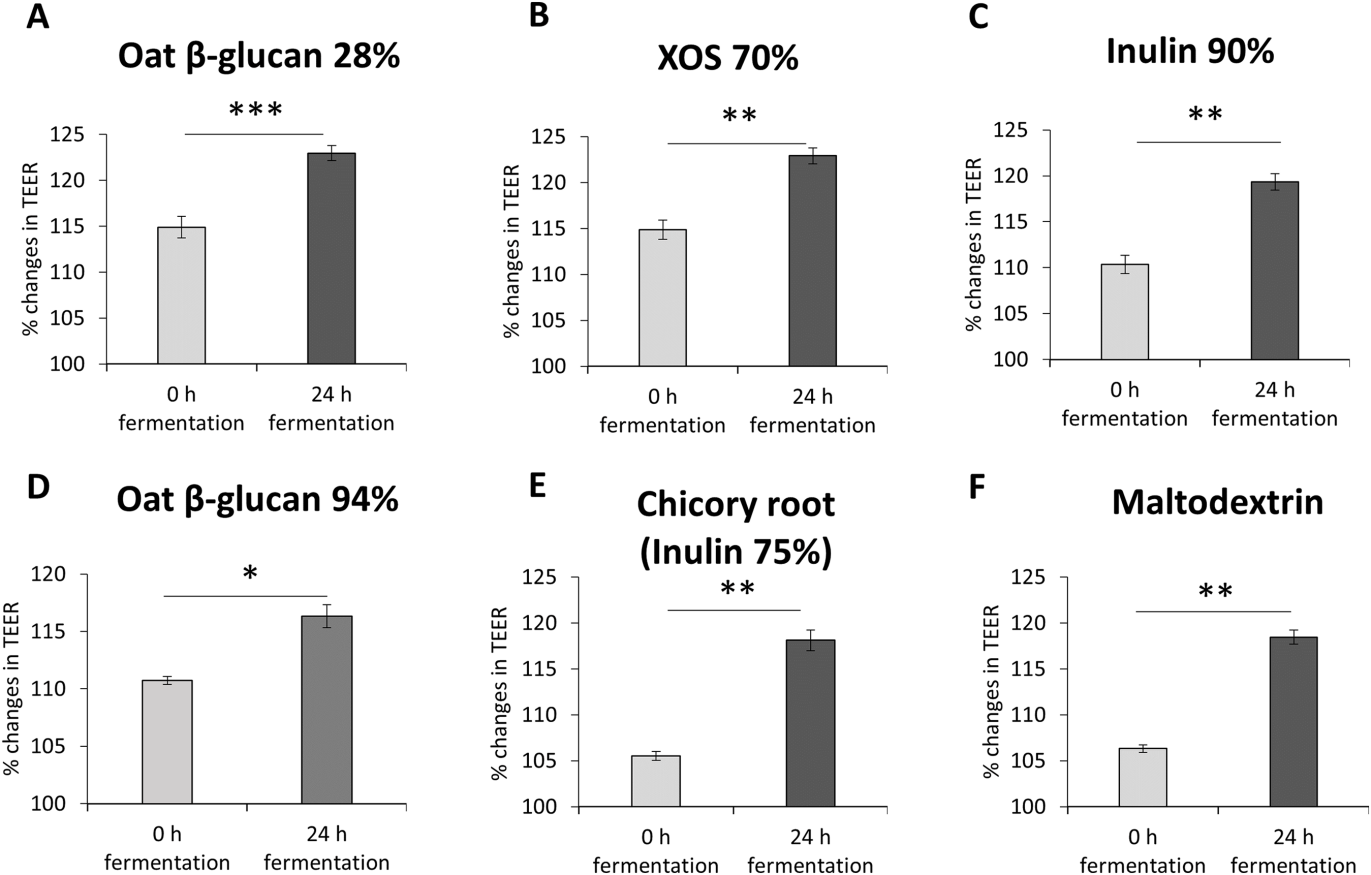
(A–F) Effects of fibre fermentation supernatant on gut barrier integrity using healthy gut model. Data are expressed as percentage changes in TEER value ± SEM, *n* = 18 (six technical replicates, three biological replicates). Initial TEER before treatment with fermentation supernatant was set as 100%. *P < 0.05, **P < 0.01, ***P < 0.001. 0 h: supernatants collected before fermentation; 24 h: supernatants collected after 24 h of fermentation of respective fibres.

To study the impact of oat β-glucan 28%, oat β-glucan 94%, inulin 90%, dried chicory root containing 75% inulin, XOS 70% and maltodextrin on epithelial barrier function, TEER was measured in a co-culture of absorptive enterocytes (Caco-2 cells) and mucus-secreting goblet cells (HT29-MTX-E12 cells) (Figs. 2A–2F). TEER significantly increased (*P* < 0.001) in cells treated with supernatant from 24 h fermentation of oat β-glucan 28% compared to cells treated with supernatant collected before fermentation (0 h fermentation) (24 h: 123.0 ± 0.8, 0 h: 114.9 ± 1.2). Treatments with supernatant from 24 h fermentation of dried chicory root containing 75% inulin (118.1 ± 1.1 vs 105.5 ± 0.5), XOS 70% (122.9 ± 0.9 vs 114.9 ± 1.0), inulin 90% (119.3 ± 0.9 vs 110.3 ± 1.0), oat β-glucan 94% (116.3 ± 1.0 vs 110.7 ± 0.4) and maltodextrin (118.4 ± 0.76 vs 106.3 ± 0.41) significantly increased TEER compared to supernatant collected before fermentation (0 h fermentation).

### Effects of fibres on gut barrier integrity using a basolateral induced leaky gut model

**Figure 3:**
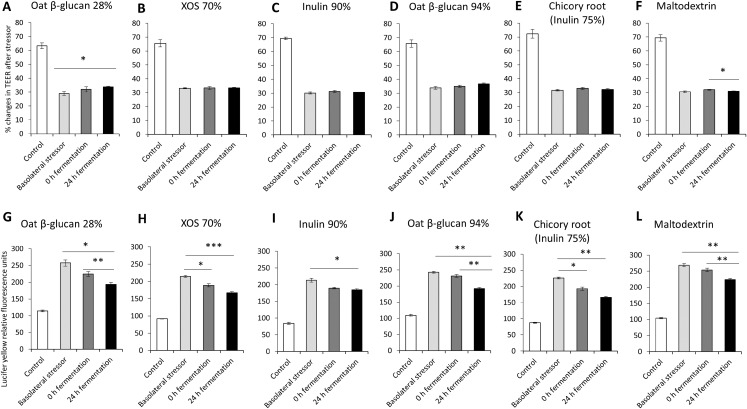
Effects of fibre fermentation supernatant on basolateral induction of leaky gut model. (A–F) Percentage changes in TEER value ± SEM, *n* = 9 (three technical replicates, three biological replicates). TEER before washing and treatment with basolateral stressor was set as 100%. (G–L) Lucifer yellow permeability, data are expressed as relative fluorescence units ± SEM. *P < 0.05, **P < 0.01, ***P < 0.001. Basolateral stressor: 5% ethanol. Control: no basolateral stressor. 0 h: supernatants collected before fermentation; 24 h: supernatants collected after 24 h of fermentation of respective fibres.

The impact of oat β-glucan 28%, oat β-glucan 94%, inulin 90%, XOS 70%, dried chicory root containing 75% inulin and maltodextrin fermentation products on tight junction development of co-cultures of Caco-2 and HT29-MTX-E12 were further studied via the basolateral induction of leaky gut by ethanol (Fig. 3). The treatment of the co-culture with ethanol as the basolateral stressor resulted in approximately 50% reduction of TEER compared to the untreated control (without stressor: 67.7 ± 3.4%, after stressor: 31.5 ± 1.9%). The supernatant of the in vitro fermentation of oat β-glucan 28% by the gut microbiota for 24 h resulted in a significant increase in TEER (*P* < 0.01) compared to the basolateral stressor control (24 h: 33.8 ± 0.5%, basolateral stressor: 29.0 ± 1.4%) (Figs. 3A–3F). There was no significant change in TEER in cells treated with 24 h fermentation samples compared to 0 h fermentation samples. TEER after treating the cells with supernatant from 24 h fermentation of XOS 70%, inulin 90%, oat β-glucan 94%, and dried chicory root containing 75% inulin was not significantly different compared to supernatant from unfermented samples (0 h fermentation) and ethanol control. In contrast, supernatant from the fermentation of maltodextrin for 24 h significantly decreased TEER compared to 0 h fermentation supernatant (24 h: 31.0 ± 0.2%, 0 h: 32.1 ± 0.3%).

The permeability of Lucifer yellow was measured to monitor the integrity of the tight junction of the co-cultures under basolateral stress (Figs. 3G–3L). We observed significant decrease of relative fluorescence units in cells treated with supernatant of 24 h fermentation of oat β-glucan 28%, oat β-glucan 94% and maltodextrin compared to cells treated with supernatant of unfermented samples (0 h fermentation), but not for inulin 90%, XOS 70%, and dried chicory root containing 75% inulin (oat β-glucan 28%: 193.5 ± 6.0% vs. 224.5 ± 7.4%; oat β-glucan 94%: 192.1 ± 3.7% vs. 230.7 ± 4.3%; maltodextrin: 223.6 ± 4.0% vs. 253.7 ± 5.5%). The supernatant of 24 h fermentation of all tested fibres resulted in a significant reduction in Lucifer yellow permeability compared to the basolateral stressor control (oat β-glucan 28%: 193.5 ± 6.0% vs. 257.5 ± 9.4%; XOS 70%: 167.0 ± 3.3% vs. 214.4 ± 2.5%; Inulin 90%: 184.7 ± 3.3% vs. 213.4 ± 5.5%; oat β-glucan 94%: 192.1 ± 3.7% vs. 242.7 ± 2.8%; dried chicory root containing 75% inulin: 166.3 ± 3.1% vs. 225.9 ± 2.9%; maltodextrin: 223.6 ± 4.0% vs. 268.4 ± 5.0%). We also observed a significant effect of the fermentation supernatant at time point 0 on reducing Lucifer yellow relative fluorescence units compared to the basolateral stressor control (*P* < 0.05) in the fermentations with XOS 70%, and dried chicory root containing 75% inulin.

### Effects of fibres on gut barrier integrity using an apical induced leaky gut model

**Figure 4:**
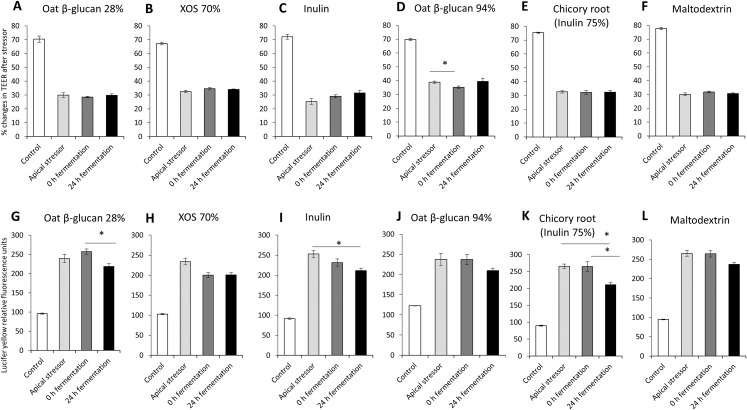
Effects of fibre fermentation supernatant on apical induction of leaky gut model. (A–F) Percentage changes in TEER value ± SEM, *n* = 9 (three technical replicates, three biological replicates). TEER before washing and treatment with apical stressor was set as 100%. (G–L) Lucifer yellow permeability, data were expressed as relative fluorescence units ± SEM. *P < 0.05. Apical stressor: Rhamnolipids. Control: no apical stressor. 0 h: supernatants collected before fermentation; 24 h: supernatants collected after 24 h of fermentation of respective fibres.

The impact of oat β-glucan 28%, oat β-glucan 94%, inulin 90%, XOS 70%, dried chicory root containing 75% inulin and maltodextrin on tight junction development of co-cultures of Caco-2 and HT29-MTX-E12 were further studied using apical induction of leaky gut by rhamnolipids (Fig. 4). Similar to the treatment with basolateral stressor, the concentration of the apical stressor was chosen such that the treatment of the co-culture resulted in approximately 50% reduction of TEER compared to the untreated control (without stressor: 72.1 ± 3.8%, after stressor: 31.6 ± 4.5%).

There was no significant impact on TEER values of cultures treated with 24 h fermentation samples of all tested fibres compared to cultures treated with samples before fermentation (0 h fermentation) and compared to cultures treated with the apical stressor control (Figs. 4A–4F).

We observed significant decrease of relative Lucifer yellow fluorescence in cultures treated with supernatant of 24 h fermentation of oat β-glucan 28% (218.8 ± 7.3% vs. 257.3 ± 6.8%) and dried chicory root containing 75% inulin (210.8 ± 7.1% vs. 265.0 ± 14.1%) compared to cells treated with unfermented supernatant (0 h fermentation) (Figs. 4G–4L). The treatment with 24 h fermentation samples (24 h fermentation) of inulin 90% and dried chicory root containing 75% inulin resulted in a significant reduction in Lucifer yellow permeability compared to the apical stressor control (Inulin 90%: 211.3 ± 6.3% vs. 252.8 ± 8.9%; dried chicory root containing 75% inulin: 210.8 ± 7.1% vs. 265.4 ± 6.8%) (Figs. 4G–4L).

### Effects of fibres on mucus production of HT29-MTX E12 cells

**Figure 5:**
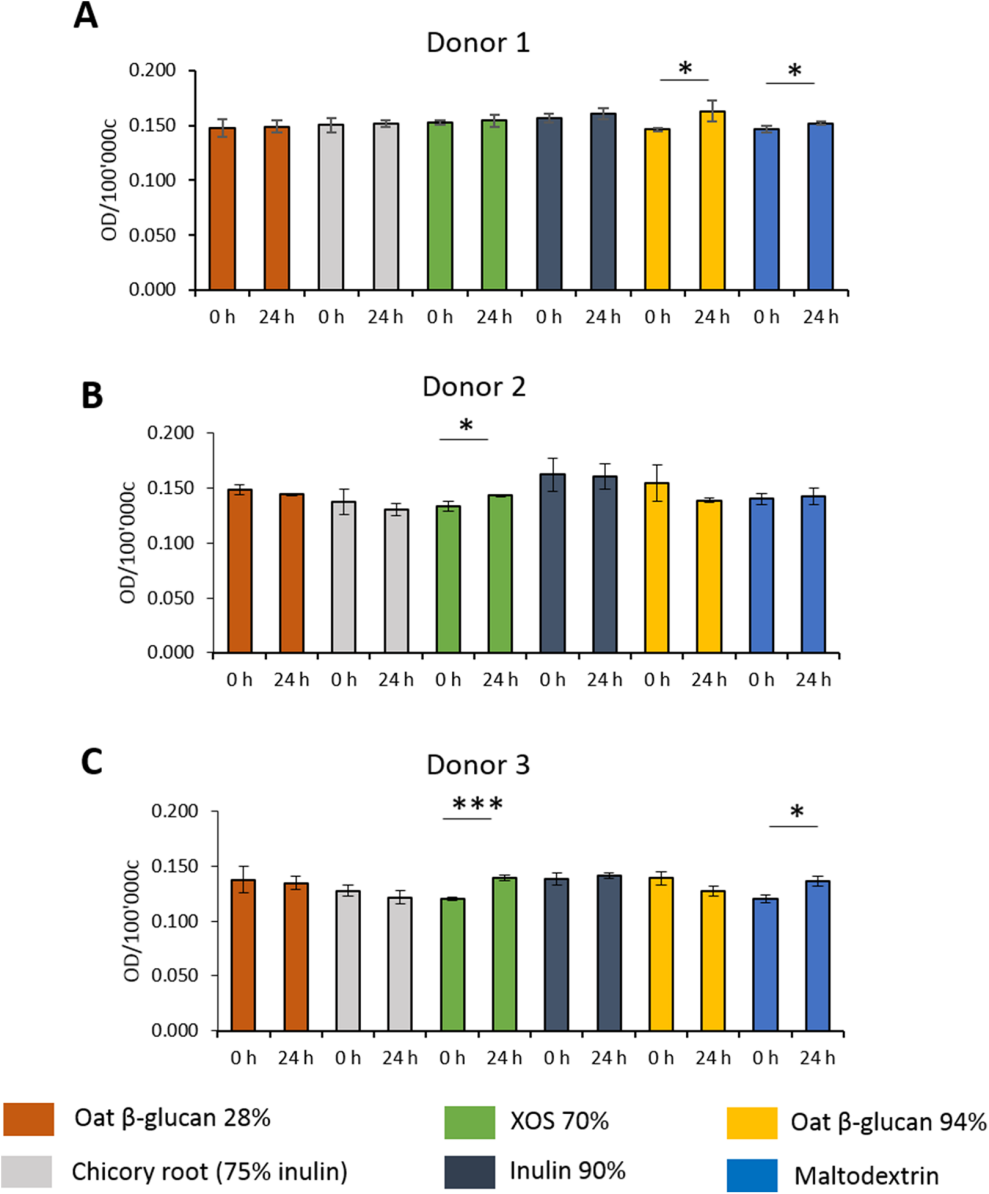
Effect of fibre fermentation supernatant on mucin production of HT29-MTX cells. (A–C) Data are expressed as normalized Alcian Blue absorbance ± SD, *n* = 3 (three technical replicates). *P < 0.05, ***P < 0.001. Control: fermentation medium.

To study the effects of fibres on mucus production, HT29-MTX-E12 cells were stained by Alcian Blue. We observed inter-individual variability in mucus production of cells treated with supernatant from fermentation of faecal samples from three donors (Figs. 5A–5C). Mucus production was significantly increased in cells treated with supernatant from 24 h XOS 70% fermentation using faecal samples from donor 2 (0.143 average 600 nm absorbance ± 0.001 vs. 0.133 ± 0.005) and donor 3 (0.139 ± 0.003 vs. 0.120 ± 0.002). Oat β-glucan 94% fermentation products significantly increased mucus production of cells treated with the supernatant from fermentation of faecal sample obtained from donor 1 (0.163 ± 0.010 vs. 0.146 ± 0.001). On the other hand, oat β-glucan 94% decreased mucus production of cells treated with 24 h fermentation supernatant from flasks inoculated with donor 2 and donor 3, albeit without statistical significance. The 24 h treatment of supernatant from maltodextrin fermentation of faecal samples from donor 1 (0.152 ± 0.001 vs. 0.147 ± 0.003) and donor 3 (0.136 ± 0.005 vs. 0.120 ± 0.004) resulted in a significant reduction in mucus production compared to time point 0.

### Effects of fibres on immunological biomarkers

**Figure 6:**
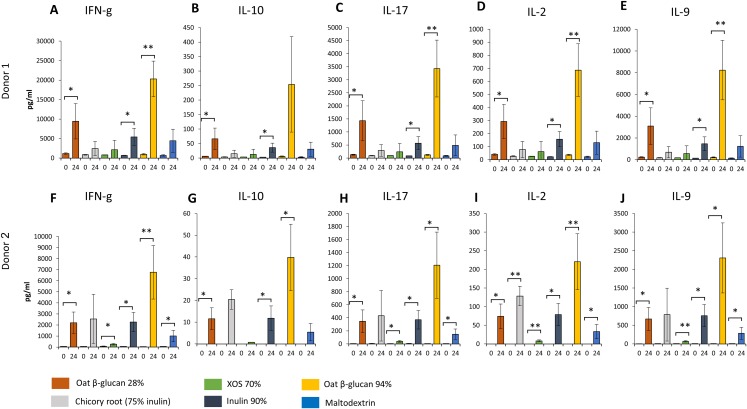
Effect of fibre fermentation supernatant on cytokine/chemokine production by HT29 cells. (A–J) Data are expressed as pg/ml ± SD, *n* = 3 (three technical replicates). *P < 0.05, **P < 0.01. Times indicated on the *x*-axis indicate fermentation duration.

Similar changes in levels of all immunological biomarkers were seen between donor 1 and donor 2 after 24 h fermentation with the tested fibre supernatants. Levels of all immunological biomarkers in samples delivered from donor 3 were below the detection levels and therefore not reported in this study. We observed in effluent from 24 h fermentation of oat β-glucan 28% using faecal samples from donor 1 (Figs. 6A–6E) and donor 2 (Figs. 6F–6J) a significant increase in IFN-γ (Donor 1: 9443.3 ± 4539.8 vs. 1134.0 ± 196.5 pg/mL, Donor 2: 2203.3 ± 962.6 vs. 52.7 ± 2.8 pg/mL, respectively), IL-10 (Donor 1: 66.4 ± 37.2 vs. 6.1 ± 0.5 pg/mL, Donor 2: 11.6 ± 5.0 vs. 0.0 ± 0.0 pg/mL), IL-17 (Donor 1: 1427.7 ± 765.7 vs. 128.0 ± 15.6 pg/mL, Donor 2: 347.0 ± 174.1 vs. 4.7 ± 0.2 pg/mL), IL-2 (Donor 1: 292.3 ± 132.0 vs. 40.4 ± 5.7 pg/mL, Donor 2: 73.9 ± 33.3 vs. 0.0 ± 0.0 pg/mL), and IL-9 (Donor 1: 3086.7 ± 1682.0 vs. 237.7 ± 27.7 pg/mL, Donor 2: 669.7 ± 308.3 vs. 5.8 ± 1.2 pg/mL) compared to timepoint 0. Levels of all immunological biomarkers were higher in 24 h fermentation samples with oat β-glucan 94% compared to other tested fibres. Levels of IFN-γ (Donor 1: 20300.0 ± 4596.7 vs. 983.3 ± 197.0 pg/mL, Donor 2: 6763.3 ± 2422.9 vs. 44.5 ± 9.8 pg/mL), IL-10 (Donor 2: 39.8 ± 15.3 vs. 0.0 ± 0.0 pg/mL), IL-17 (Donor 1: 3430.0 ± 1088.1 vs. 122.0 ± 17.1 pg/mL, Donor 2: 1207.3 ± 508.2 vs. 3.5 ± 0.0 pg/mL), IL-2 (Donor 1: 687.0 ± 202.1 vs. 36.4 ± 5.3 pg/mL, Donor 2: 220.3 ± 75.1 vs. 0.0 ± 0.0 pg/mL), and IL-9 (Donor 1: 8243.3 ± 2743.2 vs. 216.0 ± 42.8 pg/mL, Donor 2: 2306.7 ± 938.3 vs. 6.5 ± 1.8 pg/mL) found in 24 h oat β-glucan 94% fermentation samples were significantly higher compared to time point 0 in both donors. We observed in effluent from 24 h fermentation of inulin 90% using faecal samples from both donors a significant increase in IFN-γ (Donor 1: 5430.0 ± 2208.7 vs. 678.7 ± 41.5 pg/mL, Donor 2: 2263.3 ± 868.4 vs. 62.8 ± 7.8 pg/mL, respectively), IL-10 (Donor 1: 35.8 ± 15.6 vs. 2.8 ± 0.4 pg/mL, Donor 2: 11.8 ± 5.6 vs. 0.0 ± 0.0 pg/mL), IL-17 (Donor 1: 571.3 ± 248.8 vs. 87.0 ± 4.7 pg/mL, Donor 2: 370.3 ± 142.6 vs. 6.1 ± 2.5 pg/mL), IL-2 (Donor 1: 157.2 ± 58.5 vs. 22.7 ± 1.8 pg/mL, Donor 2: 78.8 ± 29.7 vs. 0.0 ± 0.0 pg/mL), and IL-9 (Donor 1: 1463.0 ± 640.7 vs. 147.7 ± 11.0 pg/mL, Donor 2: 758.3 ± 296.2 vs. 6.1 ± 1.9 pg/mL) levels compared to time point 0.

Levels of IFN-γ, IL-17, IL-2, and IL-9 found in 24 h XOS 70% and maltodextrin fermentation samples were significantly higher compared to time point 0 in donor 2. Significant higher concentration of IL-2 were detected from 24 h fermentation samples of dried chicory root containing 75% inulin compared to time point 0 in donor 2.

## Discussion

The role of probiotics in regulating epithelial tight junction proteins and to protect the epithelial barrier has been reported in vivo (Ulluwishewa et al., 2011). However, little is known about the impact of prebiotics on gut barrier and immunity functions. In this study we aim to compare the fermentation products of the commercially available fibres on parameter of the gut barrier integrity and the immune system. Our results demonstrate the beneficial effect of the fermentation products of commonly used fibres to promote the integrity of the epithelial barrier, using an in vitro cellular model of Caco-2 and HT29-MTX-E12 cell lines. We further presented the effect of the fermentation products of prebiotic fibres in the production of inflammatory cytokines.

In the current study, the supernatant from fermentation of all tested fibres led to an increased TEER in a cellular model mimicking a healthy gut. Oat β-glucan 28% was most effective in this model, probably due to the fact that it is a complex mixture of dietary fibres with additional nutritional compounds such as insoluble fibres, proteins, unsaturated fatty acids, vitamins and minerals. During experimental induction of intestinal permeability in vitro (leaky gut model), oat β-glucan 28%, oat β-glucan 94% and maltodextrin reduced Lucifer yellow permeability. Moreover, oat β-glucan 28% and dried chicory root containing 75% inulin played a protective role in the reversing the gut permeability induced by an apical stressor. The mechanisms by which prebiotics promote epithelial barrier integrity are not well established. In a recent study, the fermentation of oat β-glucan 28% and oat β-glucan 94%, which supernatants were used in this study, promoted propionate production after 24 h (Carlson et al., 2017). This result suggests that the fermentation of prebiotics strengthens the intestinal barrier in healthy and leaky gut condition by the production of SCFAs. Butyrate has been shown to exert positive effects on several human organs including the maintenance of the barrier function in the gut through its stimulation effect on mucus production, expression of tight-junction proteins and antibacterial peptides as well as leading to lower colonic oxidative stress (Riviere et al., 2016). The effect on mucosal layer and barrier integrity is particularly important as its changes directly affect human health by restricting the access of bacteria and their undesired metabolites to reach the systemic circulation. Such defect in barrier function has been described in Inflammatory bowel disease (IBD) (Barbosa & Rescigno, 2010). Indeed, propionate and acetate has been shown to increase TEER and reduce Lucifer yellow permeability in rat caecum, T84 and Caco-2 cell monolayers (Suzuki, Yoshida & Hara, 2008). Furthermore, a recent study in colitis mice demonstrated the effect of sodium propionate to ameliorate dextran sulphate sodium-induced colitis and improve intestinal barrier function by inhibiting the decrease of ZO-1 and occludin expression in the colonic tissue (Tong et al., 2016). While the importance of butyrate in the maintenance of the intestinal barrier has been well established (Macfarlane & Macfarlane, 2011), the role of propionate needs to be explored further in future studies. Intriguingly, a recent study indicates the direct effect of prebiotics to enhance epithelial barrier integrity to protect against pathogen-induced barrier disruption in Caco-2Bbel monolayers and in duodenal organoids (Wu et al., 2017). Whether the tested fibres in this study have such direct effect remains to be elucidated.

In this study, we reported a remarkable similar impact of oat β-glucan 28% and oat β-glucan 94% fermentation supernatant in modulating immunological biomarkers. Both enhanced IFN-γ, IL-10, IL-17, IL-2, and IL-9 levels in both donor 1 and 2. Oat β-glucan 94% fermentation products led to a higher increase of cytokines and chemokines compared to oat β-glucan 28% fermentation products, suggesting that metabolites from oat β-glucan fermentation play an important role in modulating cytokine and chemokine productions. It is widely accepted that pro- and anti-inflammatory signals are both important for a balanced immune response (Buckley & Rast, 2017; Clarke & Chintanaboina, 2018; Koboziev et al., 2015; Kotredes, Thomas & Gamero, 2017; Martin-Subero et al., 2016; Pott & Stockinger, 2017; Sarrabayrouse et al., 2015; Webb & Tait Wojno, 2017). Even if the modulated cytokines may exert both pro- and anti-inflammatory properties, our results clearly show that their expression is highly regulated by the fermentation supernatant of oat β-glucan 28% and oat β-glucan 94% in two of three donors. We interpret these data such that fermentation supernatant of oat β-glucan is the active ingredient of oat β-glucan 28% for modulation of the immune parameters. We also showed that, inulin 90% fermentation products enhanced levels of the tested cytokines and chemokines in both donors. Our data is in concordance with a recent study showing that inulin 90% enhanced LPS induced IL-10 production (Liu, Gibson & Walton, 2016). On the other hand, the increase of IFN-γ by the tested fibres fermentation products in our in vitro models contradict previous result, in which prebiotic-fed mice have lower level of plasma IFN-γ (Cani et al., 2009). Sundin et al. stimulated colonic biopsies of healthy controls and post-infectious IBS patients with selected species of anaerobic commensal bacteria. The levels of several immune modulating cytokines such as interleukins differed between and healthy controls and post-infectious IBS patients providing the evidence of a dysregulated immune response to the commensal gut bacteria in post-infectious IBS patients (Sundin et al., 2015). Our results may be relevant to examine the therapeutic potential of prebiotics in the management of inflammatory bowel disease, IBS, and infection.

The intestinal epithelium is covered by a mucus layer which serves as a physical barrier preventing luminal bacteria from directly contacting the cell layer (Konig et al., 2016). On the other hand, defective mucus production has been involved in several immune-mediated diseases. The dysbiosis in gut microbiota is shown to play a role in the intestinal pathogenesis such as IBS and IBD in addition to extra intestinal diseases including obesity, metabolic syndrome, depression, diabetes and so on (Carding et al., 2015; Mohajeri et al., 2018). In our study, treating the goblet cells with effluent from XOS 70% and maltodextrin fermentation significantly increased mucus production. Recently, the effect of prebiotic XOS on modulating the composition of human gut microbiota by increasing bifidobacteria has been well studied in vitro and in vivo (Carlson et al., 2017; Finegold et al., 2014; Li et al., 2015; Lin et al., 2016; Moniz et al., 2016). Similarly, the growth of bifidobacteria was significantly stimulated by the fermentation of maltodextrin (Liu, Gibson & Walton, 2016). Several studies in rats shown that *B. bifidum* R0071 and a probiotic mixture called VSL#3 which contains three *Bifidobacterium* strains stimulated mucin secretin and mucin gene expression. Whether the effect of XOS and maltodextrin on mucin production seen in this study is due to metabolites produced by *Bifidobacterium* requires further investigation.

An advantage of our study in comparison to studies using only the Caco-2 cells as the model for gut-barrier is the use of the co-culture of Caco-2 and HT29-MTX-E12 epithelial cells which represent better the inner wall of the human gut in which the epithelial cells are covered by a mucus layer. One limitation of our study was the use of supernatants collected from a batch fermentation which has a short operation time of 24-h due to the rapid depletion of substrate, the accumulation of microbial metabolites and reduction of pH which can prevent further microbial activity (Payne et al., 2012; Venema & Van Den Abbeele, 2013). Such short-term fermentations might not support the establishment of a trophic chain where fermentation product from one species becomes substrate to another species. Another limitation is the lack of the quantification of tight junction proteins such as occluding, ZO-1, which could confirm the impact of fibres on gut barrier integrity and might further elucidate mechanism of actions. Hence, further study investigating prebiotics should include tight junction proteins measurement in a continuous fermentation model such as SHIME or PolyFermS models (Pham & Mohajeri, in press).

## Conclusion

Impaired gut barrier has been shown in IBS (Saulnier et al., 2013) and diarrhoea-predominant IBS (Gecse et al., 2012), however there is limited evidence of the beneficial effect of prebiotics in IBS subjects. Similarly, while present therapy in IBD mostly focuses on treating the inflammatory symptoms after gut ‘leakage,’ little is known about the potential of prebiotic to reduce paracellular leakage that contributes to the proinflammatory state (Valenzano et al., 2015). Moreover, increased intestinal permeability was found in patients with pathogenic protozoan infections (Dagci et al., 2002). The results of our study demonstrated the potential of the most commonly used fibres on strengthening the gut barrier and immunity functions. Our data suggest that the various dietary fibres affect the host physiology differently and call for studying the role of well-defined fibres to promote specific health conditions in humans.

## Supplemental Information

10.7717/peerj.5288/supp-1 Supplemental Information 1 TEER and Lucifer yellow raw data.

10.7717/peerj.5288/supp-2 Supplemental Information 2 Mucus staining raw data.

10.7717/peerj.5288/supp-3 Supplemental Information 3 Cytokine production raw data.

## Additional Information and Declarations

### Data Availability

The following information was supplied regarding data availability: The raw data are provided in the Supplemental Files.
