# Correction: The effects of fermentation products of prebiotic fibres on gut barrier and immune functions in vitro

**DOI:** 10.7717/peerj.5288/correction-1

**Published:** 2018-08-17

**Authors:** Van T. Pham, Nicole Seifert, Nathalie Richard, Daniel Raederstorff, Robert E. Steinert, Kevin Prudence, M. Hasan Mohajeri

**Affiliations:** R&D Human Nutrition and Health, DSM Nutritional Products Ltd., Basel, Switzerland

Corrigendum:

After publication, the authors noticed that the fourth author's name is written incorrectly. Robert Steinert is now corrected to Robert E. Steinert in the author list and contributions.

